# Management of Permanent Teeth in Dentigerous Cysts in Children: A Case Report

**DOI:** 10.7759/cureus.44062

**Published:** 2023-08-24

**Authors:** David Antunes, Amelie Albisetti, Margaux Fricain, Adam Cherqui, Stephane Derruau

**Affiliations:** 1 Department of Oral Surgery, University Hospital of Reims Champagne-Ardenne, Reims, FRA; 2 Department of Oral Surgery, Faculty of Dental Surgery, Strasbourg University, Strasbourg, FRA; 3 Department of Oral and Maxillofacial Surgery, Head and Neck Institute, University Hospital of Nice, Nice, FRA; 4 Department of Orthodontics, University Hospital of Reims Champagne-Ardenne, Reims, FRA; 5 BioSpecT EA7506, Faculty of Pharmacy, University Hospital of Reims Champagne-Ardenne, Reims, FRA

**Keywords:** mixed dentition, orthodontic treatment, cyst decompression, conservative treatment, unerupted teeth, dentigerous cyst, case report

## Abstract

Dentigerous cysts are the second most common odontogenic lesion, after radicular cysts. Dentigerous cysts mainly affect individuals in their second to fourth decades of life, with a slight male predominance. Because diagnosis is often late, surgical procedures like enucleation and removal of the impacted tooth misplaced are often necessary.

However, if a dentigerous cyst is detected early in a child with delayed tooth eruption, the treatment goal is to preserve and properly position the permanent tooth within the arch. In such cases, conservative approaches like cyst decompression may be considered appropriate.

We present a case of a dentigerous cyst in a 10-year-old child with delayed eruption of teeth 22 and 23. The condition was managed using decompression alone and orthosurgical traction, which facilitated the proper placement of the impacted teeth within the arch. This article emphasizes the significance of a multidisciplinary approach involving surgical and orthodontic management for dentigerous cysts in children, along with the importance of patient compliance with the treatment plan.

## Introduction

Dentigerous cysts (DC) are a type of inflammatory odontogenic cyst associated with an included or impacted tooth according to the 2017 WHO classification [1]. They represent about 20% of all odontogenic cysts, exhibit a male predominance, and have the highest incidence in the second to fourth decades of life [1]. DC are most often associated with mandibular third molars (75% of cases), followed by maxillary canines, maxillary third molars, and mandibular second premolars [2].

DC are benign lesions that encompass the crown of an unerupted tooth and can grow a very large size. Various therapeutics exist and are used with a therapeutic approach based on the patient’s age, localization of the cyst, and the tooth implicated. Conservative techniques, such as marsupialization or cystic decompression, are particularly indicated in children with immature permanent teeth contained within the DC [3]. Marsupialization was first described by Partsch in 1892 [4] who performed a large surgical window kept open by suturing the cystic membrane to surrounding soft tissues of the oral cavity, so as to promote drainage of the cyst. Otherwise, decompression takes up the principles initiated by Partsch, but drainage is achieved through a smaller mucous window held open by an adjoining device that communicates the cystic contents with the oral cavity [5]. Despite their similar nature, the objective of these techniques is the same: to keep the cystic contents open so as to promote constant drainage involving a progressive decrease in intracystic pressure and thus promote its regression and bone regeneration. When permanent teeth in children are included and repressed, the challenge itself is no longer only bone regeneration but the hope that the tooth will be able to function on the arch through favorable tooth movement and root formation.

This case report follows the CARE (2017) guidelines for case reports [6]. We present a case of a DC in a 10-year-old child located in the anterior maxillary region, affecting the eruption of the left lateral incisor and left maxillary canine. In this context, we opted for a conservative treatment approach involving decompression followed by orthodontic treatment. The challenge in this case was to manage the DC and to position the two permanent teeth on the dental arch to ensure both aesthetics and function.

## Case presentation

A 10-year-old male patient was referred to the Oral Medicine Department of the Reims University Hospital for an orthodontic consultation due to the unilateral non-eruption of the left lateral incisor. The patient had no particular medical or surgical history.

Extra-oral clinical examination showed no abnormalities, including no swelling or lymphadenopathy.

**Figure 1:**
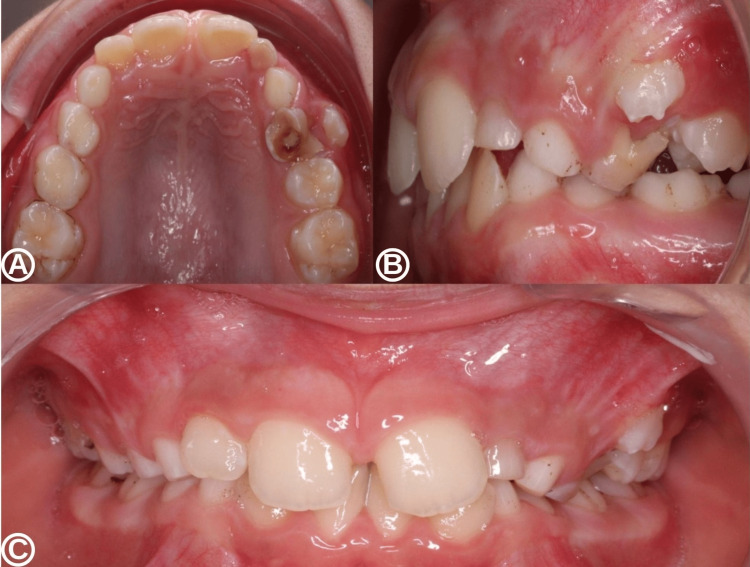
Intra-oral examination at the first surgical consultation. (A) Occlusal view of the maxilla with the persistent deciduous left incisor. (B) Lateral view of the left maxilla, focusing on the canine region. (C) Frontal view of the maxillary arch in occlusion, showing a rotated position of the left maxillary central incisor and a discrete localized swelling within the vestibule in the region of the left canine.

Intra-oral clinical examination (Figure 1) revealed the persistence of the left deciduous lateral incisor, the left deciduous canine, and the left deciduous first molar, the latter of which had a cavity and terminal mobility. The permanent left incisor was malpositioned, and a slight vestibular swelling was noted adjacent to the periapical area of the deciduous left incisor and canine in the maxilla. This swelling was painless on palpation. No signs of infection, including suppuration, were observed on palpating of the vestibular area. The cold pulp test was positive on all the teeth in the left maxillary region and the percussion test was physiological.

**Figure 2:**
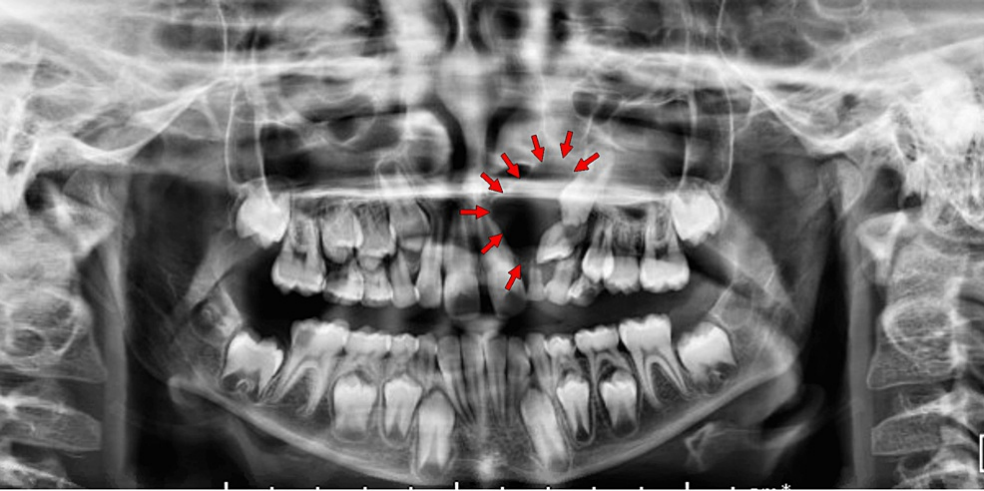
Initial dental panoramic radiography. Large radiolucent lesion in the canine region with left deciduous lateral incisor and canine impacted.

**Figure 3:**
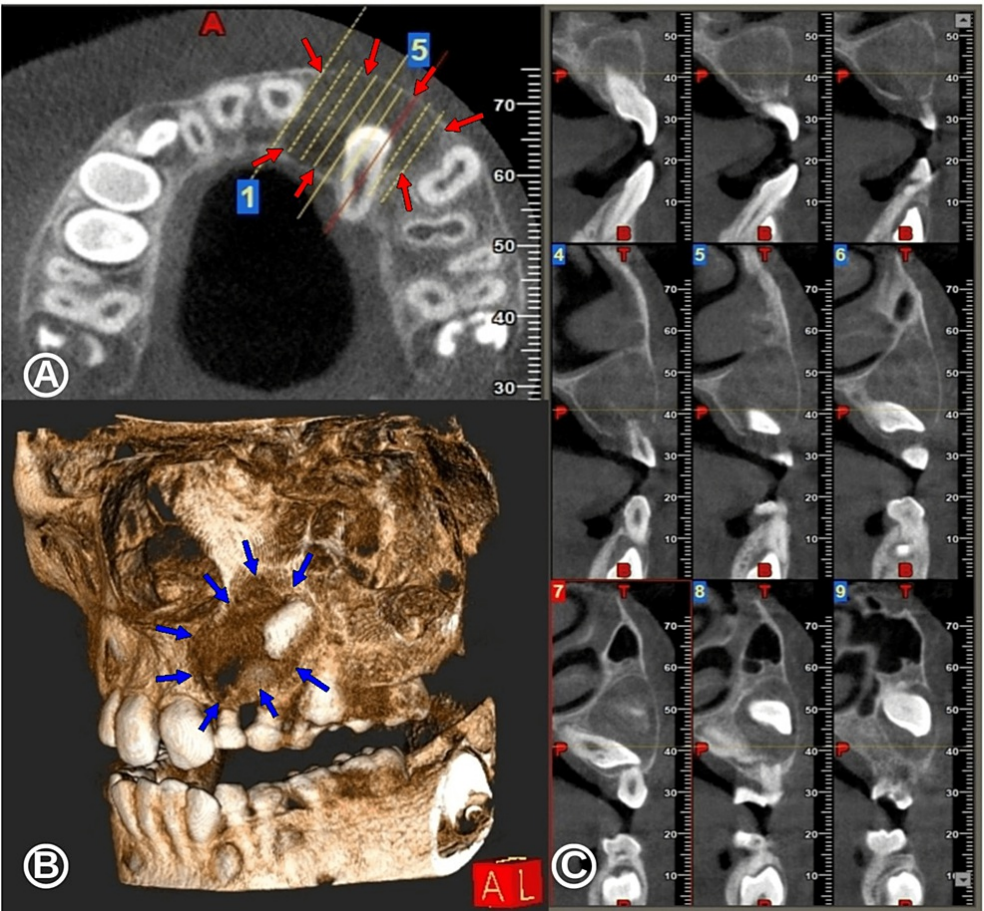
Cone-beam computed tomography (CBCT) images from the first surgical consultation. (A) Axial view showing a unilocular radioclear lesion encompassing the crown of the permanent left maxillary canine. (B) Three-dimensional CBCT reconstruction centered on the maxillary left canine region with the dentigerous cyst. (C) Series of CBCT images in sagittal view illustrating the extent of the dental cyst, which encompasses the crowns of the permanent maxillary left lateral incisor and canine.

At first, panoramic radiography was performed, uncovering a radiolucent image, homogeneous, unilocular, well-delimited, and located within the left maxillary permanent incisor and canine, both enclosed in an ectopic position (Figure 2). The permanent left central incisor was displaced with apical mesiorotation and coronal distorotation. Further, a cone-beam computed tomography (CBCT) three-dimensional image was conducted, revealing a radioclear image measuring about 21 mm x 17 mm x 24 mm, inserting itself into both the neck of the permanent left lateral incisor and canine included (Figure 3).

**Figure 4:**
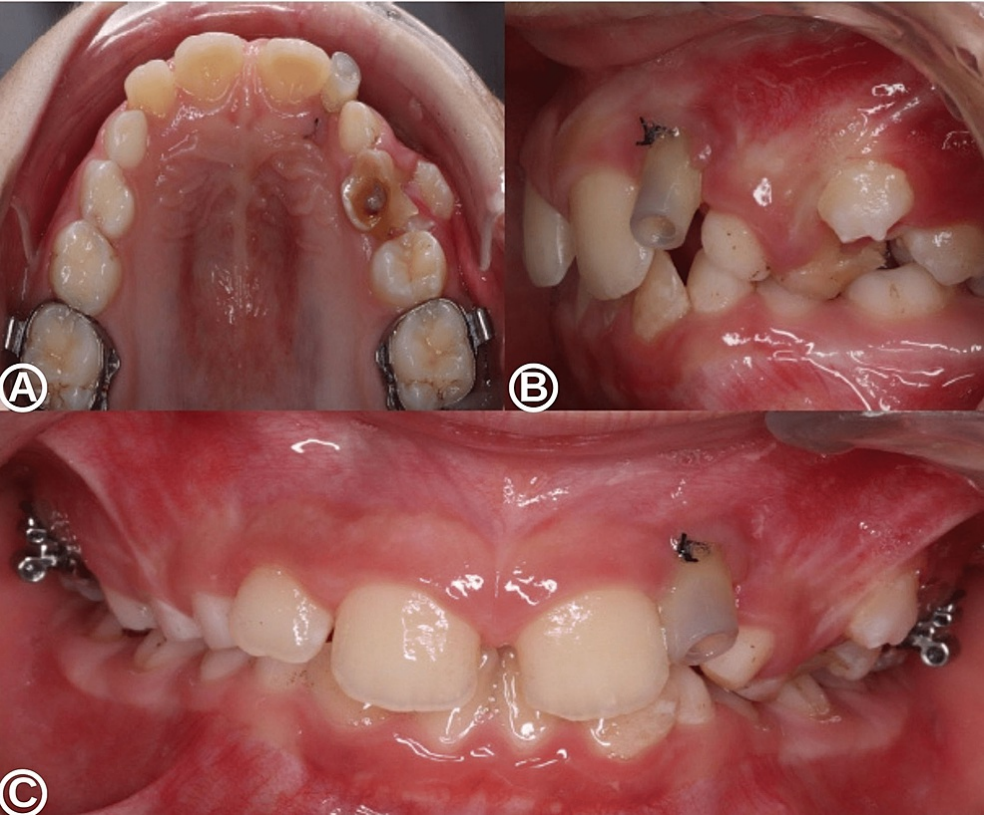
Intra-oral situation during the process of cyst decompression. Occlusal (A), left lateral (B), and frontal (C) views focused on the left canine region. A silicone drain was sutured with non-absorbable sutures at the site of the temporary left lateral incisor's socket.

The deciduous left lateral incisor, in direct communication with the cystic lesion, of this 10-year-old child was then extracted and a sample of the cystic membrane was obtained for histological analysis. A silicone tube was placed in the cystic cavity through the socket of the extracted tooth in order to decompress the cyst (Figure 4). Amoxicillin 500mg to be taken twice a day for seven days, and an analgesic medication containing 500mg of paracetamol has been prescribed. Additionally, an antiseptic mouthwash based on 0.12% chlorhexidine gluconate to realize twice a day for two weeks has been prescribed. Subsequently, the patient was advised to perform daily rinses of the cystic cavity using a syringe, physiological serum, and povidone-iodine through the drain.

**Figure 5:**
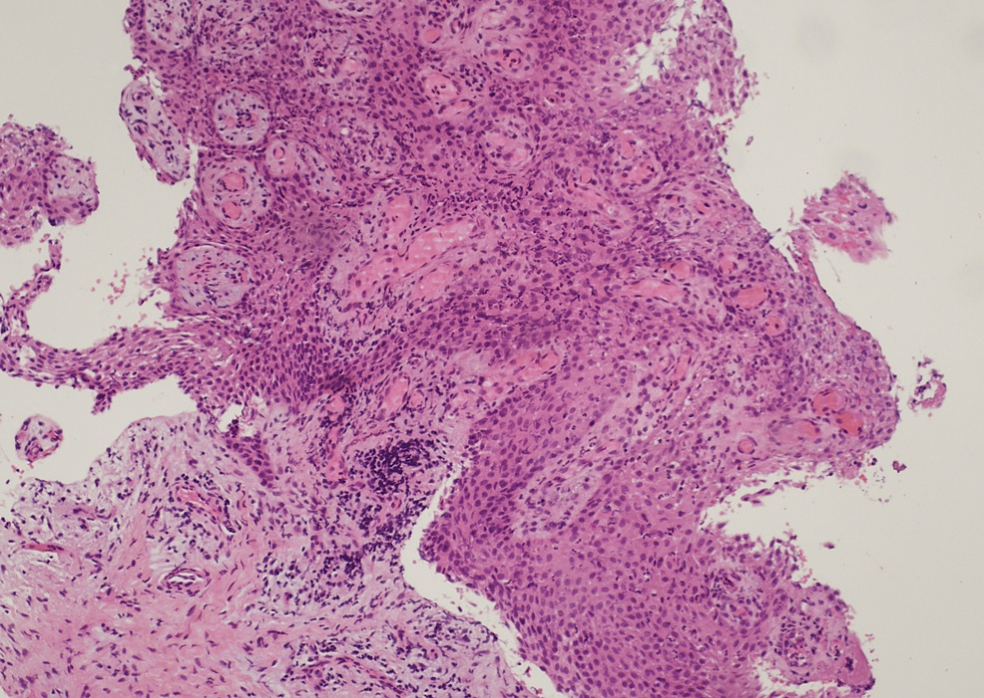
Histological section of cyst biopsy. Saffron hematoxylin and eosin (HES) stain, x10 magnification.

Anatomopathological analysis revealed a non-keratinized squamous epithelium of variable thickness containing inflammatory remodeling with polynuclear cells and lymphocytes, without any suspicious character. This appearance is compatible with the diagnosis of an inflamed DC, given the context of impacted teeth (Figure 5).

**Figure 6:**
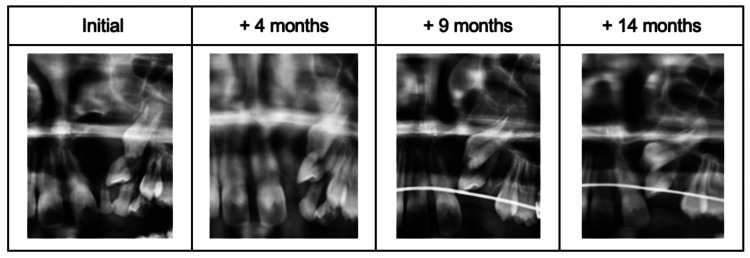
Radiographic follow-up of the dentigerous cyst in the left canine region.

In order to avoid mesialization of the left maxillary posterior region, a Nance arch was placed with regular orthodontic appointments. After four months of cystic decompression, the drain was removed; the left deciduous canine and the left deciduous first molar were extracted. Clinical and radiographic follow-up, including dental panoramic radiography, were performed and showed progressive regression of the cyst and progression in the coronary direction of the permanent left lateral incisor and maxillary canine with root apexification (Figure 6).

**Figure 7:**
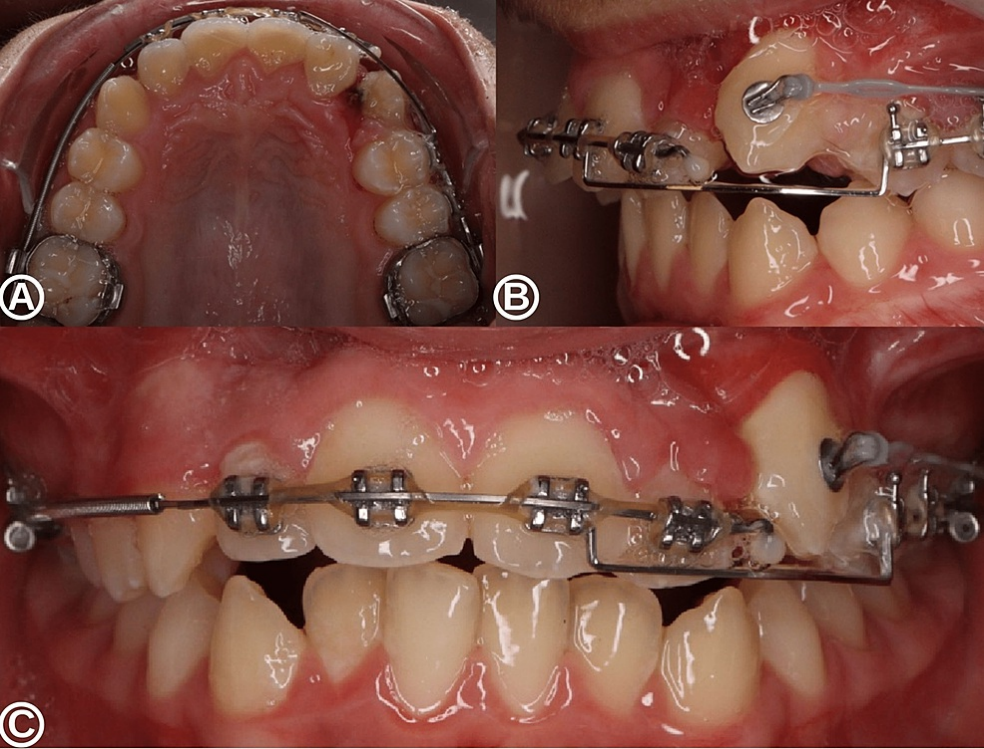
Intra-oral situation with orthodontic treatment at four years after the beginning of decompression. Occlusal (A), lateral (B), and frontal (C) views centered on the left canine region with the permanent maxillary left lateral incisor and canine in place within the dental arch by orthodontic treatment.

The permanent maxillary lateral incisor continued to erupt naturally over the next nine months without orthodontic intervention. However, orthodontic-surgical traction was required for the permanent left maxillary canine after one year of cyst decompression. The left maxillary canine became fully functional four years after the initiation of cyst decompression (Figure 7).

**Figure 8:**
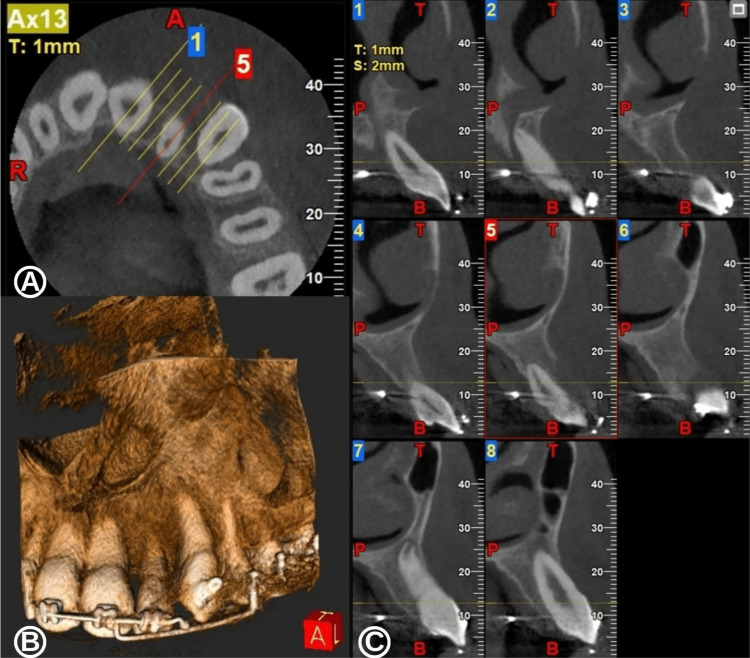
Cone-beam computed tomography (CBCT) images four years after the beginning of decompression surgery. (A) Axial view revealing the position of the roots of the permanent left lateral incisor and canine and the absence of a radioclear image. (B) Three-dimensional CBCT reconstruction showing tooth position and bone healing in the maxillary canine region. (C) Series of CBCT images in sagittal view validating the complete regression of the dentigerous cyst and the successful apexification of the permanent left lateral incisor and canine.

Finally, with a conservative approach combining a minimal surgical technique by decompression and orthodontic treatment, the DC appeared to have been completely eliminated by the placement of the causal impacted tooth within the dental arch (Figure 8).

## Discussion

DC arise from the dental follicle of an unerupted or developing teeth, attached at the cementoenamel junction. They represent the second most common odontogenic lesion after radicular cysts [1]. However, the frequency of odontogenic cysts in children is relatively low. It has been estimated that approximately 4% to 9% of DC occur in the first decade of life [7].

The pathogenesis of DC is related to pressure from an erupting tooth on the follicle, potentially obstructing circulation and leading to the accumulation of exudate between the reduced enamel epithelium and the tooth crown [8]. Managing this lesion in children requires careful consideration and may differ from adults. In fact, when a cyst affects a child and causes delayed eruption, the prognosis for the permanent teeth is often compromised, which is a source of anxiety for both the child and the parents. Therefore, proposing a reliable technique for cyst removal while preserving the immature teeth involved is of interest.

According to Berretta et al. [3], both cystic decompression and marsupialization are effective techniques in managing benign cysts such as dentigerous and radicular cysts, as well as more aggressive lesions like keratocysts and unicystic ameloblastomas. Before considering these conservative techniques, a correct diagnosis through anatomopathological sampling is crucial. Complete surgical enucleation with the extraction of teeth related to the lesion becomes necessary in the management of aggressive lesions like ameloblastoma or keratocysts, whereas cystic decompression alone may be sufficient for DC. For the different types of cysts, decompression or marsupialization has shown no significant difference in cyst reduction [3]. Bone healing after cyst removal is a multifactorial process influenced by bone remodeling, the size of the lesion, and the anatomical structures involved [9]. Importantly, decompression appears effective in cases of DC, where resorption is more rapid than in radicular cysts or keratocysts [10]. The use of cyst decompression in children gives many advantages using a minimally invasive procedure including preserving and developing permanent tooth germ and protecting important anatomical structures [11]. Although some cases report a risk of development of ameloblastoma in situ or micro-invasive ameloblastoma or other neoplastic transformations of the DC wall, decompression techniques seem widely indicated to encourage permanent teeth to remain in the arch. The major disadvantages of this technique are the very limited histopathologic examination of the whole cyst epithelium and the longer time required for management [12].

A recent study by Nahajowski et al. [13] investigated predictive factors favoring the placement of teeth impacted by a DC on the arch. Age and stage of dental development appear to be predictive factors correlated with spontaneous dental eruption after marsupialization. Spontaneous eruption occurs at the beginning of the first decade and when root development is less than or equal to half of complete formation [14,15]. This finding could be partially explained by the greater potential for bone regeneration in younger individuals. Tooth eruption does not always occur spontaneously after decompression or marsupialization, particularly when there is insufficient space to allow eruption or when no favorable axis is available [16,17]. Additional orthodontic management might be necessary to ensure optimal permanent tooth placement on the arch.

In our patient's case, the left maxillary lateral incisor was at Nolla stage 7, while the permanent left canine was at stage 6. His young age at the time of the fortuitous discovery of the cyst, the absence of complete root edification of the impacted teeth, and his compliance with treatment were key factors that had a major impact on the prognosis of the teeth, which are now functional. The cyst decompression procedure lasted approximately four months. These results seem to be in agreement with those reported by Allon et al. [18], where the estimated mean decompression period was 7.5 months in children under 18 years old. The review proposed by Berretta et al. [3] suggests a maximum time of 23.3 months. Thus, informing patients in advance about the time required for these techniques to be effective is crucial for achieving optimal compliance, which is fundamental to treatment success [13].

In this case, follow-up every two months was carried out by both an oral surgeon and an orthodontist, enabling us to effectively manage permanent tooth placement in the arch. Close collaboration is essential to ensure that orthosurgical traction of the canine is performed at the right time, depending on its intraosseous three-dimensional position and degree of root development. It is also important to emphasize that no additional cyst enucleation surgery was required, and complete cyst recurrence was confirmed through three-dimensional imaging. A similar finding is supported by Lizio et al. [19], who also proposed that uncomplicated cystic decompression might serve as a definitive treatment for DC, eliminating the need for secondary surgical intervention, a success mirrored in our case.

A few similar cases published in the scientific literature have shown a similar approach to the management of DC in children. Takagi et al. [20] reported the case of a six-year-old patient whose DC, associated with the left maxillary second premolar, was successfully treated by decompression of the cyst alone. After an 18-month period of decompression, orthodontic treatment was undertaken, and a five-year follow-up was carried out with appropriate realignment of the teeth. There was no recurrence of the cyst and no need for further surgery. Sahin et al. [11] presented a case of a DC on the left mandibular second premolar of a seven-year-old girl. A drain was left in place for six months and at 40 months, the premolar had successfully erupted without cystic enucleation surgery. In our case, orthodontic treatment was introduced after 12 months, and the patient was followed up for 48 months, resulting in the successful placement of the permanent left canine within the dental arch.

However, long-term follow-up remains essential until the impacted tooth is properly placed in the arch. The recurrence of DC is uncommon, particularly after complete cyst removal or tooth eruption.

## Conclusions

Given its prevalence, knowledge of the different treatments for DC, depending on the patient's age and dental training, is fundamental. In children, a conservative approach by marsupialization or decompression will always be favored in the first instance so as to encourage the maintenance of the affected permanent teeth within the dental arch. Close collaboration between orthodontists and oral surgeons is essential.

Nevertheless, a histological analysis remains essential to avoid misdiagnosing cysts or more aggressive tumors such as keratocyts, ameloblastomas, or other aggressive tumors. Finally, to ensure the effectiveness of the treatment, strict compliance and hygiene must be maintained throughout the treatment course as a guarantee of success.
